# Non-verbal communication questionnaire: a measure to assess effective interaction

**DOI:** 10.3389/fpsyg.2024.1409675

**Published:** 2025-01-08

**Authors:** Maryam Khan, Sana Zeb, Rabia Batool, Agata Gasiorowska

**Affiliations:** ^1^Faculty of Psychology, SWPS University, Wroclaw, Poland; ^2^School of Economics, Quaid-i-Azam University, Islamabad, Pakistan; ^3^Department of Psychology, Muslim Youth University, Islamabad, Pakistan

**Keywords:** discouraging cues, encouraging cues, effective communication, human interaction, non-verbal communication

## Abstract

In five studies, we document the development and validation of the Non-verbal Communication Questionnaire (NVCQ). This eight-item measurement tool assesses how people perceive non-verbal cues across two dimensions of effective communication. These two dimensions, encouraging and discouraging non-verbal cues, are based on Khan and Zeb's (2021) version of the 10-part model of non-verbal communication. Study 1 reports the development of the NVCQ and provides initial support for the factorial structure of the measure in a Pakistani sample. Studies 2 and 3 confirmed the factorial structure and demonstrated the construct validity of the NVCQ. A preregistered Study 4 confirmed the factorial structure in a Polish sample, and provided additional support for the construct validity of the measure, while Study 5 demonstrated its adequate test–retest reliability. We conclude that the NVCQ is a psychometrically sound instrument for assessing effective communication that incorporates non-verbal aspects in every domain of life, from clinical to research settings.

## Introduction

Behavior analysts and researchers often quote management consultant Peter F. Drucker when discussing the topic of non-verbal communication, who said, “The most important thing in communication is to hear what is not being said” (Ratcliffe, 2017). As the statement makes clear, what is not said goes beyond the verbal message, but carries some weight and sometimes great importance in completing the idea. The typical non-verbal communication patterns affect every interaction in people’s daily lives and are important to the transmission and reception of what they are meant to do through effective communication. Without the component of non-verbal communication, a verbal utterance not only nullifies effective communication but also diminishes the essence of the message if it is understood differently. Therefore, we need to understand how people interpret non-verbal social cues and what we make of them (Eunson, 2012).

Non-verbal communication plays an important role in human interaction and has been studied extensively in the literature. These non-verbal cues, also known as “tells, “include facial expressions, eye movements, gestures, and body language, which can provide information about the speaker’s state of mind (Navarro, 2011). However, the effect of these cues can vary depending on the context in which they are used. For example, appropriate eye contact between speakers has been shown to positively affect communication. In contrast, a prolonged stare or lingering gaze can hinder the smooth flow of a conversation. Similarly, energetic physical cues such as nodding and smiling (Kjellmer, 2009) are generally seen as encouraging, whereas excessive movement of hands and other body parts can hinder conversation.

In addition, effective articulation of the voice and appropriate intonation can contribute to a successful communication outcome. However, the spatial distance between the speaker and the listener can also play a decisive role in communication effectiveness. The position of the speaker in relation to the listener can influence the “approach” of the tone, whether contact is made or broken (Eunson, 2012). When people meet for the first time, they form an impression of each other based on these non-verbal cues and gestures. Regardless of whether this impression is right or wrong, it influences how one thinks and how one behaves toward the other in conversation (Darioly and Mast, 2014).

Non-verbal communication is a crucial aspect of human interaction in conveying emotions, attitudes, and intentions (Navarro, 2011). It encompasses various behaviors, including facial expressions, eye gaze, gestures, paralinguistics, proxemics, and appearance cues. Facial expressions have been studied extensively as a critical component of non-verbal communication. Similarly, eye contact is an important non-verbal cue conveying interest, attraction, hostility, and the like (Bavelas et al., 2002). Gestures, such as waving, pointing, nodding, or thumbs up, can also convey information and are often culturally specific (Hostetter and Alibali, 2007). Paralinguistic features such as tone of voice, inflection, pitch, volume, and silence differ from the actual language of spoken words and have a unique impact on the sensitivity of the message (Knapp et al., 2012). Proxemics, or personal space, is a socially conditioned and normative aspect of non-verbal behavior that determines the appropriate distance one should maintain when speaking with another person. Body postures, movements, touch and appearance cues, such as clothing and hairstyle, can influence how the message is judged and interpreted (Eunson, 2012).

In a gender-oriented analysis, Sud (2011) concluded that women exhibit higher levels of competence in both recognizing and expressing non-verbal cues. A meta-analysis by Hall (1978), including 75 studies, concluded that women performed better in interpreting non-verbal cues. It is noteworthy, however, that although gender differences are manifested, they are not of substantial magnitude, as demonstrated by Hall, who suggested that other factors, particularly those of a personal and interpersonal nature, may be partly responsible for this ability (Hall, 1979, as cited in Sud, 2011).

Similarly, many studies have explored the role of age in sensitivity to or recognition of non-verbal cues and have shown that these abilities increase with age (Feldman and Tyler, 2006; Lieberman et al., 1988). However, sensitivity to non-verbal cues was reported to vary across the lifespan, with improved sensitivity to and recognition of non-verbal cues from childhood through adulthood, but declining to some extent in older age (Feldman and Tyler, 2006). Another study concluded that with age both the use and understanding of non-verbal communication generally increase (DePaulo and Rosenthal, 1979).

The 10-part model of non-verbal communication (also known as the visual model of non-verbal communication), developed by Eunson and Eunson (1987), emphasizes key elements that can influence how a message is received and interpreted. These 10 elements include (1) facial expressions, (2) eye contact, (3) gestures, (4) posture, (5) proximity, (6) haptics (touch), (7) appearance, (8) voice quality, (9) silence, and (10) time. Previous research has shown that these elements are cross-cultural and can convey important information to others. For example, facial expressions can express emotions such as happiness, anger, or sadness, while gestures can convey emphasis or intention. Proximity and touch can signal intimacy or aggression, and the quality of voice and silence can convey an emotion or indicate the power dynamic in a conversation. Understanding the role of non-verbal communication can be beneficial in various contexts, from personal relationships to business interactions. By paying attention to these cues and responding appropriately, you can improve your communication skills and increase the accuracy of your messages (Dickson et al., 2003).

All in all, this 10-part model of non-verbal communication summarizes the essential non-verbal cues, including facial expressions, gestures, posture and eye contact, etc., as well as the associated functioning of these aspects of everyday interaction. Eunson’s model has also shown that these cues are universally applicable across cultures and have the potential to convey intentions, emotions or even status dynamics such as the power of the speaker. Building on this model, Khan and Zeb (2021) went a step further and categorized these cues as determinants of effective communication, such as maintained eye contact, voice modulation, etc., and barriers to effective interaction, such as prolonged staring, longer distances, and similar aspects. This experiment paved the way for the development of an instrument to measure the role of non-verbal cues in ordinary interactions that depend on such contrasting brackets of unsaid but explained and constructed aspects of verbal messages.

In this project, we have set ourselves the goal of developing a psychometrically reliable and user-friendly instrument for measuring non-verbal communication between people. Despite the importance of non-verbal communication emphasized by the 10-part model, the lack of psychometrically sound assessment tools is an obstacle in this area. Therefore, we aimed to address this gap by developing a measurement tool based on Khan and Zeb's (2021) version of the 10-part model that captures the role of non-verbal cues and how individuals perceive them.

In summary, non-verbal communication is a multifaceted phenomenon that plays a crucial role in human interaction (Tabensky, 2008). Also, researchers pointed that while planning a study on NVC, the lack of potential tool was the basic trouble (Khan and Zeb, 2021); insufficiency of scales measuring non-verbal competence (Puertas-Molero et al., 2022) and deficiency of non-verbal assessment tools (Abbas and Khan, 2023) highlighted the dire need to tackle this issue first. The present research aimed to develop a psychometrically sound instrument for measuring non-verbal communication between individuals, thus filling a gap in the existing literature. The instrument can measure various aspects of non-verbal communication, including facial expressions, eye gaze, gestures, paralinguistics, proxemics, and appearance-based cues, and contribute to a better understanding of this crucial aspect of human communication.

## Study 1

In Study 1, we generated the pool of items for the NVCQ, verified its dimensional structure, and provided an internal consistency estimation of the measure. We assumed that the NVCQ would include two distinct aspects of non-verbal communication, beliefs about encouraging non-verbal cues (ENVCs) and discouraging non-verbal cues (DNVCs). We operationalized them based on Khan and Zeb’s (2021) version of the 10-part model of non-verbal communication, defining ENVCs as the potential for noticing elements of conversation commonly referred to as “cues” or “signals” that facilitate or reinforce the meaning of the message, while DNVC includes attempts to register or focus on cues that may interfere with conversation and make it difficult for others to understand or grasp the topic of conversation. We have attempted to frame the statements in simple language such that people from different backgrounds can easily understand them, i.e., “appropriate eye contact makes it easy for me to connect with someone/speaker/teacher or energetic bodily clues keep the conversation lively.” Furthermore, we tried to create a relatively short scale convenient for large-scale data collection. With this in mind, we invited 37 Pakistani students from a bachelor’s in computer science program, aged 19–24 (*M* = 22.8, *SD* = 1.77; 24 men, 13 women) to participate in a semi-structured interview session reflecting on DNVCs and ENVCs they found to be influential elements in conversation other than the verbal aspect of communication. They also shared their thoughts and perceptions about those elements. The interviews were not recorded, but the interviewers were taking notes concerning the students’ statements. After reviewing its content, we generated an initial pool of 53 statements.

To assess the content validity of the questionnaire, we presented the draft of the measure to two academics (with psychology master’s degrees) together with the definitions of the scale and subscales and asked for their independent opinion reflecting if the items correspond to the scale/subscale (rated 1) or not (rated 0). As a result, only nine items were deemed suitable and retained. These statements were then shared again with two other colleagues with the task to assess comprehensiveness. As a result of their comments, we revised the wording of some statements, dropped unnecessary words, and removed one statement that did not closely reflect the construct. Thus, at the end of this phase, we had eight face-valid items for the NVCQ, with five statements capturing the ENVCs and three statements covering the DNVCs. The items covered essential non-verbal cues including eye-contact, continued staring, bodily gestures (nodding/pointing), smile, voice quality (pitch/tone variation), and distance of speaker with an appropriate display for an effective message as explained by the participants (see Table 1). The Gunning fog index of 13.55 represented the satisfactory level of the measure in terms of its readability that anyone with 13 years of formal education can easily understand (i.e., high school level).

**Table 1:** Psychometric properties of the non-verbal communication questionnaire: factor loadings of items in Study 1 (*N* = 187), Study 2 (*N* = 202), Study 3 (*N* = 378), and Study 4 (*N* = 334).

Items	(Study 1)			(Study 2)			(Study 3)			(Study 4)		
Statements	M 1	M 2	M 3	M 1	M 2	M 3	M 1	M 2	M 3	M 1	M 2	M 3
Appropriate eye contact makes it easy for me to connect with someone/speaker/teacher	0.531	0.531	0.523	0.532	0.532	0.525	0.514	0.514	0.509	0.579	0.579	0.643
Continues staring (by the speaker/teacher) is a hurdle in effective communication	0.505	0.505	0.491	0.511	0.511	0.494	0.512	0.512	0.492	0.321	0.321	0.534
Energetic bodily clues keep the conversation lively	0.779	0.779	0.766	0.793	0.793	0.782	0.797	0.797	0.787	0.674	0.674	0.601
Nodding, pointing, thumbs up et cetera are required to keep the conversation going	0.770	0.770	0.757	0.772	0.772	0.762	0.776	0.776	0.768	0.529	0.529	0.527
Smiling start of a speaker (teacher in a classroom) has a role to create an affirmative environment to learn	0.833	0.833	0.832	0.843	0.843	0.842	0.842	0.842	0.841	0.602	0.602	0.671
Monotony of voice makes the conversation boring	0.834	0.834	0.734	0.810	0.810	0.704	0.786	0.786	0.689	0.564	0.564	0.293
Variation in the pitch of voice keeps life in a talk	0.761	0.761	0.763	0.762	0.762	0.763	0.750	0.750	0.752	0.644	0.644	0.577
It’s difficult to enjoy a conversation with a person at a larger distance from the listener/learner	0.672	0.672	0.585	0.671	0.671	0.574	0.661	0.661	0.571	0.391	0.391	0.366

Factor loading for all the studies are given as Model 1, 2 and 3 = M1, M2, and M3, respectively.

Considering our theoretical model, we initially expected that the items would converge into a second-order factor indicating beliefs about non-verbal communication. We, therefore, tested the proposed model of the NVCQ. To do this, we administered an eight-item measure to the sample of Pakistani adults, and we tested three competing models of the NVCQ: (1) a model with beliefs about encouraging and discouraging dimensions as two correlated but separate factors; (2) a model with beliefs about encouraging and discouraging dimensions as first-order factors, and a total score indicating convictions about effectiveness to non-verbal cues as a second-order factor; and (3) a model with a unidimensional structure of the measure covering non-verbal communication cues. We demonstrated that the model for the two-factor structure fits the data better, and we confirmed it in the subsequent studies.

### Participants and procedure

The sample included 187 individuals from the general population residing in Islamabad and Rawalpindi, Pakistan, aged 15–45 years (*M* = 21.64, *SD* = 3.68; 98 men, 89 women). After giving informed consent, participants were provided with the eight-item NVCQ and indicated their response on each item on a scale from 1 (*strongly disagree*) to 7 (*strongly agree*). The order of items was not randomized.

### Results and discussion

We tested the structure of the NVCQ using confirmatory factor analysis (CFA) with a maximum likelihood estimation method with bootstrapped errors (1,000 repetitions) in Jamovi. The first model, with beliefs about encouraging and discouraging cues as two correlated but separate factors, fit the data well in the light of most indices, χ^2^/df = 2.34, RMSEA = 0.084; 90% CI [0.052, 0.117]; GFI = 0.98, CFI = 0.96, TLI = 0.94, indicating the two-factor structure of the scale. As demonstrated in Table 1, the standardized factor loadings for all items were significant, indicating a moderate or strong relationship between the items and the first-order latent factor (*β*s > 0.50, *p*s < 0.001). The correlation between the two factors was strong and significant, *r* = 0.85, *p* < 0.001. The second model, with beliefs about encouraging and discouraging cues as first-order factors, and a total score indicating convictions about the effectiveness of non-verbal communication as a second-order factor, had a similar fit to the data, χ^2^/df = 2.47, RMSEA = 0.089; 90% CI [0.056, 0.122]; GFI = 0.98, CFI = 0.95, TLI = 0.93. The standardized factor loadings for all items were significant (*β*s > 0.50, *p*s < 0.001), as well as the factor loadings for the two dimensions (*β*s > 0.92, *p*s < 0.001). Finally, the fit for the model with a unidimensional structure of the measure covering non-verbal communication cues was worse than for the two previous models, χ^2^/df = 3.06, RMSEA = 0.105; 90% CI [0.075, 0.135]; GFI = 0.98, CFI = 0.93, TLI = 0.91. In this model, the standardized factor loadings for all the items were significant but slightly lower than in the previous models (*β*s > 0.49, *p*s < 0.001). Factor loading for all three models are reported in Table 1, while discrimination indices for the items are reported in Supplementary Table S1. In sum, initial analyses suggested that the two-factor structure of the scale with separate ENVC and DNVC dimensions emerged as more promising, and therefore, we decided to confirm it in further testing.

## Study 2

After we initially established the structure of the NVCQ in Study 1, the aim of Study 2 was (1) to confirm the adequacy of the two-dimensional structure of the NVCQ with a different sample; (2) to provide information about its internal consistency; and (3) to find out whether age and gender differences exist. Since prior research had shown that with age, both the use and understanding of non-verbal communication generally increases (DePaulo and Rosenthal, 1979), we expected a similar trend in different age groups. Similarly for gender differences, no established trends were reported, so in this study we explored how the beliefs or perception of non-verbal cues can differ among men and women.

### Participants and procedure

The sample included 202 Pakistani students aged 15–60 years (*M* = 21.71, *SD* = 4.37; 104 men, 98 women) from Islamabad. After giving informed consent, participants completed the eight-item NVCQ.

### Results and discussion

#### Structure of the scale

We retested the proposed factor structure of the scale using a generalized least-squares CFA with a maximum likelihood estimation method with bootstrapped errors (1,000 repetitions) in Jamovi, which yielded a good fit for the two-factor model, χ^2^/df = 2.21, RMSEA = 0.077; 90% CI [0.046, 0.110]; GFI = 0.953, CFI = 0.969, TLI = 0.955, confirming the factor structure and providing the additional evidence for the structure of the scale. As demonstrated in Table 1, the standardized factor loadings for all items were significant, indicating a moderate or strong relationship between the items and the first-order latent factor (*β*s > 0.51, *p*s < 0.001). The correlation between the two factors was strong and significant, *r* = 0.85, *p* < 0.001. The second model, with ENVCs and DNVCs as first-order factors, and a total score indicating convictions about the effectiveness of non-verbal communication as a second-order factor, had a similar fit to the data, χ^2^/df = 2.12, RMSEA = 0.082; 90% CI [0.11, 0.05]; GFI = 0.95, CFI = 0.96, TLI = 0.94. The standardized factor loadings for all items were significant (*β*s > 0.51, *p*s < 0.001), as well as the factor loadings for the two dimensions (*β*s > 0.92, *p*s < 0.001). Finally, the fit for the model with a unidimensional structure of the measure covering non-verbal communication cues was worse than for the two previous models, χ^2^/df = 1.83, RMSEA = 0.099; 90% CI [0.07, 0.12]; GFI = 0.93, CFI = 0.94, TLI = 0.92. In this model, the standardized factor loadings for all the items were significant but slightly lower than in the previous models (*β*s > 0.49, *p*s < 0.001).

Again, as a whole, the attempt suggested that the two-factor structure of the scale with separate ENVC and DNVC dimensions emerged as more promising, and therefore we decided to use it for further testing. Factor loadings for all three models are reported in Table 1, while the discrimination indices for the items are reported in Supplementary Table S1. Also, the scale exhibited a fairly good internal consistency as a whole (*ω* = 0.87, *M* = 27.91, *SD* = 11.86), as well as regarding their two factors, the ENVCs comprised of five items (ω = 0.86, *M* = 16.55, *SD* = 8.18) and the DNVCs having three items (ω = 0.70, *M* = 11.35, *SD* = 4.78).

#### Age and gender differences

Contrary to our expectation, no significant association (*r* = 0.040, *p* = 0.571) emerged for age and total NVCQ scores or for its factors, ENVCs (*r* = 0.034, *p* = 0.632) and DNVCs (*r* = 0.042, *p* = 0.557). Furthermore, the NVCQ yielded some gender differences where men scored significantly higher than women on the NVCQ in general, as well as on the ENVC and DNVC subscales (Table 2).

**Table 2:** Gender differences in the non-verbal communication questionnaire in Studies 2 and 3.

	Measures	Men		Women			
		*M*	*SD*	*M*	*SD*	*t*	Cohen’s *d*
Study 1	NVCQ	30.34	12.51	24.95	10.26	3.47**	0.47
	ENVC	17.74	8.80	14.96	7.05	2.60*	0.34
	DNVC	12.60	4.80	9.99	4.32	4.21**	0.57
Study 2	NVCQ	30.80	12.84	24.84	9.90	3.70**	0.51
	ENVC	18.15	8.97	14.86	6.90	2.92*	0.41
	DNVC	12.65	4.95	9.97	4.19	4.14**	0.58

**p < 0.01, *p < 0.05; M, mean; SD, standard deviation; NVCQ, non-verbal communication questionnaire; ENVC, encouraging non-verbal cues; DNVC, discouraging non-verbal cues.

## Study 3

After obtaining evidence for the scale’s structure, the aim of Study 3 was to establish the validity of the NVCQ using the multitrait-multimethod approach (Campbell and Fiske, 1959). Thus, participants completed our NVCQ and a revised version of the Self-Consciousness Scale (SCSR; Fenigstein et al., 1975; Scheier and Carver, 1985). The SCSR is an instrument for measuring objective self-awareness (Duval and Wicklund, 1972), built on the assumption that individuals enter a state of self-consciousness when they become the object of their thoughts. This state of self-consciousness includes three key facets of the construct: (1) private self-consciousness, referring to the internal state of a person’s thoughts; (2) public self-consciousness, concerning the external state of a person’s thoughts, or the understanding of the effect of one’s presence on others and (3) social anxiety, described as a public-pertaining version of the SCSR that includes apprehensions around being evaluated by others or doubtfulness about a desirable self-presentation before others, making it closer to the tendency of being sensitive to non-verbal cues during conversation (Schlenker and Leary, 1982).

Luan and Chen (2021) documented empathy as a moral emotion to facilitate behavioral change including cognitive and effective elements. They reported two studies’ findings that indicated a significant association between private self-consciousness and empathic concerns, thus advocating that knowledge of one’s own internal states guides people to understand others’ internal states or enables perspective-taking. Also, Magrì (2022) postulated that empathy is closer to “social sensitivity” in terms of understanding the feelings and states of others, whereas Isohätälä et al. (2021) concluded social sensitivity is an individual’s capability to read, understand, and address verbal as well as non-verbal communication in terms of appropriate social behavior. Thus, we hypothesized a positively significant relationship between private self-consciousness and sensitivity to non-verbal cues among participants. In contrast to this, a high public self-awareness has been found consistently congruent with a higher involvement in impression management (Fenigstein et al., 1975). Consistent with Wine (1971) and Hartman (1983), excessively self-focused behavior may impede individuals from paying attention to others while maintaining their own impression. Therefore, we proposed a significant negative relationship between public self-consciousness and sensitivity to non-verbal cues among participants.

Also, Hope and Heimberg (1988) presented social anxiety as an outcome of being highly self-conscious and self-focused. Furthermore, Silvia et al. (2006) postulated facial expressions as the strongest indicator of the feelings and intentions of others, like approval or liking, stemming from a happy face and approval or dislike from an angry face. This tendency of recognizing others’ emotions is scientifically termed “emotional sensitivity” (Friedman et al., 2003). Some studies have reported a direct association between social anxiety and emotional sensitivity, as socially anxious beings generally express high emotional sensitivity toward facial expressions (Frenkel and Bar-Haim, 2011). Also, socially anxious individuals were found to interpret neutral or ambiguous stimuli or expressions as threatening or negative (Bell et al., 2011). Thus, we hypothesized a negative relationship between social anxiety and sensitivity to non-verbal cues among participants.

### Participants and procedure

The sample included 220 Pakistani students aged 15–60 years (*M* = 21.81, *SD* = 4.31; 115 men, 105 women) from Islamabad. Participants completed a set of questionnaires including the NVCQ and the SCSR after giving informed consent. To assess self-consciousness, we used the revised version of the 22-item SCSR (Scheier and Carver, 1985). The scale (Cronbach’s *α* = 0.82, *M* = 44.01, *SD* = 10.13) measures the objective state of one’s self-awareness with three specific dimensions presented as subscales: (1) private self-consciousness (2) public self-consciousness and (3) social anxiety.

### Results and discussion

#### Structure of the NVCQ

First, we retested the proposed factor structure of the scale. We conducted a generalized least-squares CFA with a maximum likelihood estimation method with bootstrapped errors (1,000 repetitions) in Jamovi, which yielded a good fit for the two-factor model, χ^2^/df = 2.68, RMSEA = 0.087; 90% CI [0.058, 0.117]; GFI = 0.95, CFI = 0.95, TLI = 0.93. In Table 1, the standardized factor loadings for all items were significant, indicating a moderate or strong relationship between the items and the first-order latent factor (*β*s > 0.51, *p*s < 0.001). The correlation between the two factors was strong and significant, *r* = 0.85, *p* < 0.001. The second model, ENVCs and DNVCs as first-order factors, and a total score indicating convictions about the effectiveness of non-verbal communication as a second-order factor, had a similar fit to the data, *χ*^2^/df = 2.83, RMSEA = 0.091; 90% CI [0.062, 0.121]; GFI = 0.98, CFI = 0.95, TLI = 0.93. The standardized factor loadings for all items were significant (*β*s > 0.51, *p*s < 0.001), as well as the factor loadings for the two dimensions (*β*s > 0.9, *p*s < 0.001). Finally, the fit for the model with a unidimensional structure of the measure covering non-verbal communication cues was worse than for the two previous models, χ^2^/df = 3.33, RMSEA = 0.103; 90% CI [0.076, 0.131]; GFI = 0.95, CFI = 0.93, TLI = 0.91. In this model, the standardized factor loadings for all the items were significant but slightly lower than in the previous models (*β*s > 0.49, *p*s < 0.001). The factor loadings for all three models are reported in Table 1, while discrimination indices for the items are reported in Supplementary Table S1.

In sum, our results suggested that the two-factor structure of the scale with separate ENVC and DNVC dimensions emerged as relatively more promising, and therefore we decided to use it in further testing. We tested the internal consistency of the two factors and the total scores on the NVCQ and demonstrated that the reliability of the total score as well as the ENVC factor was very good, respectively (*ω* = 0.87, *M* = 27.77, *SD* = 11.78, and ω = 0.85, *M* = 16.41, *SD* = 8.11). The reliability of the DNVC factor came out lower (ω = 0.68, *M* = 11.35, *SD* = 4.75).

#### Construct validity analyses

Correlations between the NVCQ and the subscales of the SCSR are presented in Table 3. As hypothesized, we found a significant positive relationship between beliefs about non-verbal communication and private self-consciousness. Also, both ENVC and DNVC subscales were found to have a significant positive relationship with it, providing convergent validity evidence, whereas a non-significant relationship was observed for beliefs about non-verbal communication and public self-consciousness, with similar results for that dimension on the NVCQ. Last, contrary to our assumption, beliefs about non-verbal communication had a significantly positive rather than negative relationship with social anxiety. Furthermore, only the ENVC subscale scores exhibited a significant positive relationship with social anxiety, and a non-significant result appeared for DNVCs and social anxiety, supporting the discriminant validity of the measure.

**Table 3:** Correlations between the NVCQ, SCSR (Study 3: *N* = 220), ESQ, and TIPI (Study 4: *N* = 334) measures.

Measures	NVCQ	ENVC	DNVC
Private self-consciousness	0.234**	0.221**	0.203**
Public self-consciousness	0.066	0.068	0.048
Social anxiety	0.191**	0.250**	0.047
Healthy emotionality	0.234**	0.221**	0.203**
Outlook	0.067	0.158**	−0.089
Resilience	0.009	0.054	−0.06
Social intuition	0.316**	0.370**	0.133**
Self-awareness	0.210**	0.280**	0.036
Sensitivity to context	−0.020	0.032	−0.091
Attention	−0.129*	−0.052	−0.201**
Openness to experience	0.200**	0.236**	0.082
Agreeableness	0.171**	0.215**	0.049
Extraversion	0.030	0.084	−0.059
Emotional stability	−0.027	0.020	−0.087
Conscientiousness	0.102*	0.158**	−0.013

**p < 0.01, *p < 0.05; NVCQ, non-verbal communication questionnaire; ENVC, encouraging non-verbal cues; DNVC, discouraging non-verbal cues.

#### Age and gender differences

We found the same results for age and gender difference as in Study 2 (Table 2).

## Study 4

Having established the structure of the NVCQ measure in the Pakistani samples, the aim of Study 4 was: (1) to confirm the adequacy of the structure of the NVCQ in another culture, and (2) to further test the validity of the scale using the multitrait-multimethod method. Everything for this study was pre-registered first, and this time, we asked our participants to complete the NVCQ together with the Emotional Style Questionnaire (Kesebir et al., 2019) and the 10-Item Personality Inventory (TIPI; Gosling et al., 2003).

The Emotional Style Questionnaire (ESQ; Kesebir et al., 2019) covers six dimensions of healthy emotional life, having theoretical underpinnings in the neuroscience of emotions: outlook, resilience, social intuition, self-awareness, sensitivity to context, and attention. Outlook measures the degree to which a person’s positive emotions are persistent over time. Resilience assesses one’s ability to recover from negative emotions. Social intuition captures one’s ability to recognize non-verbal signals or cues such as reading body language, facial expression, tone, and making inferences about the mental states of others. Self-awareness reflects the ability to perceive one’s emotions based on body signals and includes sensitivity to one’s internal states. Sensitivity to context measures one’s emotional and behavioral responses in the light of the social environment. Attention refers to one’s ability to block out emotional distractions and maintain focus on the task. Importantly, especially social intuition, self-awareness, sensitivity to context, and attention seem related to non-verbal communication: social intuition involves the ability to correctly interpret and understand non-verbal signals and cues; sensitivity to context make it more reasonable to interpret what is happening in the situation; attention brings the focus on details of the interaction; while self-awareness includes sensitivity to internal states, referencing the ability to register subtle aspects while communicating. Therefore, we expected a positive relationship between the NVCQ and its subscales and the four aforementioned subscales of the ESQ. Also, we did not expect the correlations between the NVCQ and the scores on the resilience and outlook subscales of the ESQ, irrelevant to non-verbal communication skills.

In addition, although some studies suggested a relationship between the Big Five personality dimensions and non-verbal communication (Jensen, 2016), to the best of our knowledge, the direct association between beliefs or the understanding of non-verbal cues and personality traits had not been tested yet. However, Rosenthal et al. (1979) found that individuals who scored high on the Profile of Non-verbal Sensitivity (PONS) also scored high on interpersonal sensitivity, extraversion, and related traits. Later research challenged the idea that introverts are more sensitive to non-verbal communication than extraverts (Seiser, 1982). However, the author found no support for this hypothesis and made the alternative assumption that introverts are generally better conditioned than people with high extraversion because they have a higher arousal level for moderating positive and negative stimuli.

Agreeableness, undoubtedly a dimension of interpersonal behavior related to the quality of social interaction, was found to be a sound predictor of empathic concern (Melchers et al., 2016), an other-oriented disposition related to helping others in need or feeling responsible and concerned for their well-being (Mooradian et al., 2011). Moreover, openness, especially paying attention to one’s inner feelings, is close to the cognitive facet of empathy, which entails understanding others’ internal states like their thoughts, feelings, and intentions (Magalhães et al., 2012).

In sum, we hypothesized that individuals scoring higher on traits such as openness, extraversion, and agreeableness may have increased sensitivity to non-verbal cues and a greater ability to understand the internal states of others (Magalhães et al., 2012).

Emotional instability also reflects inappropriate levels of emotional arousal (Song and Shi, 2017), associated with an increased sensitivity to negative social cues (Hopkins et al., 2021; Salemink and van den Hout, 2010; Vinograd et al., 2020). This led to the conclusion that individuals high in neuroticism are more able to recognize subtle traces of negative cues than individuals low in neuroticism. Conversely, Mykytyuk et al. (2021) reported that emotional instability leads to an inability to both recognize the emotional states of others and pay attention to details, thereby reducing emotional sensitivity toward others. Given the mixed results concerning neuroticism and no results on the relationship between non-verbal cues and conscientiousness, we had no specific expectations regarding these associations. Also, we preregistered the study hypotheses, sample size, exclusion criteria, and analyses on https://aspredicted.org/5HR_CRX.

### Participants and procedure

We arbitrarily assumed that our sample should include 350 participants. Imagining potential attrition due to failed attention checks, we recruited 401 Polish students enrolled in the SONA system at a private university to complete an online survey in exchange for academic credit points. Sixty-seven students did not provide a valid response to our attention checks and, in line with the preregistered criteria, were therefore excluded from data analysis. The final sample comprised 334 participants aged 18–54 years (*M* = 26.41, *SD* = 8.55; 48 men, 285 women, one “other/prefer not to answer”).

After giving informed consent and completing the demographic form, participants were asked to complete the NVCQ, ESQ, and TIPI scales in English. The order of questionnaires was counterbalanced and all items were randomized.

#### Emotional style questionnaire

To assess the healthy emotionality of participants, we used the 24-item ESQ (Kesebir et al., 2019), having six dimensions.

#### Ten-item personality inventory

To assess the Big Five personality traits, we used the TIPI (Gosling et al., 2003), measuring extraversion, agreeableness, consciousness, emotional stability, and openness to experience.

### Results

#### Structure of the NVCQ

We conducted a CFA with a maximum likelihood estimation method in Jamovi, which yielded a good fit for the two-factor model, χ2/df = 1.87, RMSEA = 0.048; 90% CI [0.022, 0.072]; GFI = 0.98, CFI = 0.96, TLI = 0.95. The standardized factor loadings for all items were significant, indicating a moderate or strong relationship between the items and the first-order latent factor (*β*s > 0.32, *p*s < 0.001). The correlation between the two factors was strong and significant, *r* = 0.85, *p* < 0.001. The second model, with ENVCs and DNVCs as first-order factors, and a total score indicating convictions about the effectiveness of non-verbal communication as a second-order factor, had a similar fit to the data, χ^2^/df = 1.97, RMSEA = 0.051; 90% CI [0.025, 0.075]; GFI = 0.99, CFI = 0.96, TLI = 0.94. The standardized factor loadings for all items were significant (*β*s > 0.32, *p*s < 0.001), as well as the factor loadings for the two dimensions (*β*s > 0.90, *p*s < 0.001). Finally, the fit for the model with a unidimensional structure of the measure covering non-verbal communication cues was worse than the two previous models, χ2/df = 1.83, RMSEA = 0.047; 90% CI [0.021, 0.070]; GFI = 0.98, CFI = 0.96, TLI = 0.95. In this model, the standardized factor loadings for all the items were significant but slightly lower than in the previous models (*β*s > 0.29, *p*s < 0.001). The factor loadings for all three models are reported in Table 1, while discrimination indices for the items are reported in Supplementary Table S1.

In sum, initial analyses suggested a two-factor structure^1^ of the scale with separate ENVC and DNVC dimensions (see Table 1). Also, the scale exhibited a fairly good internal consistency (*ω* = 0.74, *M* = 41.21, *SD* = 6.48) on the whole, and lower internal consistency concerning its factors: ENVC (ω = 0.73, *M* = 26.78, *SD* = 4.46) and DNVC (ω = 0.43, *M* = 14.43, *SD* = 3.00).

#### Validity testing

We sought to obtain construct validity evidence for our measure using the ESQ (Kesebir et al., 2019). The ESQ consists of social intuition, self-awareness, sensitivity to context, attention, outlook, and resilience subscales. We found a positive correlation between NVCQ scores and scores on social intuition and self-awareness—a piece of evidence for convergent validity of the scale. Conversely, divergent validity evidence was apparent in the lack of significant relationships observed in the beliefs about non-verbal cues and the outlook subscale, and we found similar results for the resilience subscale. As predicted, we found a significant positive correlation between beliefs about non-verbal communication and social intuition, with consistent findings for both NVCQ dimensions (ENVC, DNVC). Similarly, we observed a significant positive relationship between NVCQ scores and self-awareness scores. However, when examining subscales, only beliefs about ENVC exhibited a positive correlation with self-awareness, while we found a non-significant relationship for beliefs about DNVC.

Contrary to our expectations, no significant relationship emerged between the NVCQ total scores and its subscales, with the scores on the sensitivity to social context subscale. Also in contrast to our assumptions, a significant negative correlation was discovered between beliefs about non-verbal communication (NVC) and attention, as well as between beliefs about DNVC and attention. In line with our hypotheses, no relationship appeared between beliefs about NVC and outlook. Nevertheless, an interesting pattern emerged when examining the dimensions separately: Beliefs about ENVC showed a significant positive relationship with outlook, while DNVC did not display a significant correlation. Also, no support was found for the association between beliefs about NVC and resilience. These findings contribute to our understanding of the construct validity of the NVCQ.

Furthermore, we examined the relationship between the total scores on the NVCQ and the scores on the TIPI. In line with our hypotheses, we found a positive correlation between beliefs about NVC and the personality traits of openness and agreeableness. Contrary to our expectations, we found no significant correlation between NVC beliefs or its dimensions and extraversion. Furthermore, while NVC beliefs did not significantly relate to emotional stability, we observed its positive correlation with conscientiousness. This pattern was also evident for the ENVC dimension, but not for the DNVC dimension (Table 3). These findings offer insights into the nuanced associations between NVC and Big Five personality traits.

## Study 5

Our aim for the pre-registered^2^ Study 5 was to assess the test–retest reliability of the NVCQ after establishing its construct validity in studies 3 and 4. For this purpose, participants who completed the NVCQ in the previous study were contacted after 6 months to complete the questionnaire again.

### Participants and procedure

We invited 334 participants from Study 4 to complete the NVCQ 6 months after the first wave using the SONA system. Fifty-four engaged in this study within the subsequent 2 weeks (10 men, 46 women, one “other/prefer not to answer,” age *M* = 26.35, *SD* = 8.57). As in the previous studies, participants completed the eight-item NVCQ presented in random order and answered some basic demographic questions.

### Results and discussion

We calculated the Pearson correlation between the Time 1 and Time 2 scores for overall NVCQ scores and its subscales, ENVC and DNVC. Across 6 months, the test–retest reliability coefficient for the total scores on the NVCQ was *r* = 0.70, *p* < 0.001, suggesting good reliability. The coefficients for the subscales were in the acceptable range, respectively *r* = 0.60 for ENVC and *r* = 0.57 for DNVC (both *p*s *<* 0.001). These results indicated sufficiently good reliability for the NVCQ and its subscales over the 6 months, complementing our analysis of the scale’s psychometric adequacy.

## General discussion

Based on communication science, research has created a theoretical framework for understanding the perception and influence of non-verbal cues. We aimed to create a psychometrically sound measure for research and applied settings, such as clinical, educational, and industrial settings. In four studies (total *N* = 987), we found that the NVCQ, with its eight items, has good psychometric potential and is a reliable and valid measure for assessing beliefs about effective NVC skills.

### Contributions to understanding effective communication skills

We demonstrated a significant positive relationship between the total scores on the NVCQ and both of its dimensions and the scores on the private self-consciousness and anxiety subscales of the SCSR (Self-Consciousness Scale) as the preliminary evidence of construct validity for the measure. In contrast, we did not find similar relationships between beliefs about NVC and the public self-consciousness subscale. This observation seems to contradict the insights provided by Wine (1971) and Hartman (1983) that publicly self-focused individuals enact excessively self-focused behavior that impairs attention to others while maintaining their impression. However, individuals with high levels of self-consciousness in public may prioritize their impression management over paying attention to the non-verbal cues of others.

Contrary to our assumption, we found that social anxiety was positively (and not negatively) associated with the total scores on the NVCQ. Some models suggest that socially anxious individuals avoid negative social cues to manage their anxiety and reduce negative social interactions (Buckner et al., 2010). Instead, our results are consistent with the idea that individuals with social anxiety still actively perceive non-verbal cues, although the way they respond to these cues may differ from individuals without social anxiety (Christensen et al., 1980). We consider this a contribution of our study, demonstrating a relationship between the two constructs. We encourage future researchers to further explore and promote the interrelated concepts presented here for the development of effective communication skills.

Next, we sought validity evidence for our NVCQ with the ESQ (Emotional Style Questionnaire), covering six dimensions of a healthy emotional life (Kesebir et al., 2019). We found a significant positive relationship between self-awareness and the ENVC dimension, but not with the DNVC dimension. This observation aligns with Ickes et al. (1973), who suggested that the theory of objective self-awareness could temporarily impact one’s self-esteem, defined as a measure of perceived social value or the extent to which an individual feels valued by their surroundings. Low self-esteem is characterized by sensitivity to even subtle hints of being valued (Weisbuch et al., 2009).

Moreover, we did not find any relationship between beliefs about NVC and sensitivity to social context, which is a measure of one’s emotional and behavioral responses in light of the social environment (Kesebir et al., 2019). This lack of the hypothesized relationship might stem from the fact that a high focus on surroundings might enhance impression management (Fenigstein et al., 1975), reducing one’s tendency to register non-verbal information. Furthermore, contrary to our expectations, we found a negative relationship between attention and beliefs about NVC attention, stemming from its negative association with DNVC. Kesebir et al. (2019) defined attention as the ability to filter out emotional distractions and to stay focused on a specific task, which, in our case, may be understood as an ability to screen-out the one aspect of the social environment to be attended to, ignoring other aspects that are not important, such as irrelevant non-verbal cues.

Subsequently, we interpret our result for ENVCs and outlook as indicating that sustaining positive emotions is associated with or manifested through ENVCs, rather than being centered on discouraging cues. Moreover, as expected, we found no evidence for the link between beliefs about NVC and resilience, defined as the capacity to recover from negative emotions.

Moreover, in our attempt to find evidence for the relationship between NVCQ and TIPI scores, we advanced the claims about personality traits and NVC (Magalhães et al., 2012). We did not find any support for our assumption regarding the relationship between beliefs about NVC and extraversion, which might be addressed in light of the mixed findings for both extraversion and introversion as associated with the perception of non-verbal cues requiring further exploration for clarity, as discussed in the literature (Seiser, 1982). Also, in our exploratory motives, we found no significant results for beliefs about NVC and emotional stability. Some research has shown that emotional instability reflects an inappropriate level of emotional arousal (Song and Shi, 2017) that can hinder one’s ability to notice details like non-verbal cues as guided by the negative relationship of neuroticism with social intuition and attention (Kesebir et al., 2019). Also, since Jensen (2016) concluded “mixed findings” for the association between neuroticism and non-verbal cues, we thus expect additional research to assess the independent nature of traits like social anxiety (as stated earlier) and whether neurotic people recognize both positive or negative non-verbal cues or just negative cues, or to strengthen the direct association between the two constructs.

We also found a significant positive relationship between beliefs about NVC and conscientiousness, and beliefs about ENVC and conscientiousness. However, we found no results for any beliefs about DNVC and conscientiousness. This is not only an addition to the literature but also supports the assertion that conscientiousness is a tendency strongly associated with emotions related to attentiveness, also an aspect of positive affect (Fayard et al., 2012) and recognized as having a bigger predictive role in empathy (Melchers et al., 2016). Thus, based on the nature of such tendencies to be aligned with higher social sensitivity, one can expect and address the connection of the two variables.

Finally, we tested the NVCQ’s potential measurement invariance across the Polish and Pakistani samples (see Supplemental Materials) and found evidence for configural (the similar two-factor model of the construct) and metric invariance (similar item-loadings), as well as scalar invariance (similar item-intercepts). Therefore, our scale has the potential for cross-country comparisons in these two cultures. The use, influence, and interpretation of non-verbal cues are genuinely prevalent and processed in both cultures, but there might be a distinction in the meanings associated with certain cues. For example, nods, glances, and pointing might have had different meanings in the Pakistani sample (Ali et al., 2021) than in the Polish sample (Biernacka, 2020). For instance, direct eye contact is expected in Polish culture and considered a sign of honesty and trustworthiness, whereas in Pakistani culture it is considered rude and a sign of arrogance or an attempt to seek validation. In short, a non-verbal message that has a specific meaning in one society can have a completely different meaning in another (Matsumoto, 2006). However, it is important to note that the Polish sample were entirely psychology students, whereas the Pakistani sample represented a general-student population. Psychology students might be more oriented, aware, and inclined to notice non-verbal cues than the general population. Thus, our results need to be treated with caution in different cultural contexts.

Effective communication is undeniably a crucial aspect in life, from personal to professional interaction. Scholars like Jarolmen (2018) have highlighted the importance of non-verbal cues and interpreting emotions in the therapeutic process, while Bambaeeroo and Shokrpour (2017) considered this essential for educators. In addition, charismatic or transformative leaders often use non-verbal behavior to convey supportiveness and gain professional success, which is a prominent factor in organizational effectiveness (Darioly and Mast, 2014). Although numerous studies have examined communication skills training using various training strategies and interventions, they have mostly focused on language and overlooked the non-verbal component. One example is using mindfulness to promote “supportive communication,” a method that requires a keen awareness of both verbal and non-verbal messages—a concept Wyer and Adaval (2003) referred to as “cognitive indexing.” The mindful description or careful expression of emotions enhances the exchange and interpretation of communication cues for both the speaker and the listener. Jones and Hansen (2015) suggested that mindfulness helps people recognize the appropriate communicative action for given situations. Adopting our NVCQ, researchers can plan evidence-based intervention studies to change the beliefs about non-verbal cues or the perception of certain non-verbal cues like eye contact. Being mindful of non-verbal cues and their potential interpretation can facilitate communication, prevent unintended messages, and help people behave cautiously around certain elements that depend on context. Therefore, investigations of a change or shift in communication dynamics, like beliefs about non-verbal cues, might be a promising avenue to explore and advance attempts at effective communication.

### Study limitations and future research

The NVCQ was developed to be a short and practical easy-to-answer self-report instrument that might be widely used in research, academic, clinical, and industrial settings. However, self-assessment tools have limitations and must be supplemented by objective measures, whenever possible. The subjective self-assessment aspect inherent to the NVCQ is the obvious limitation of the scale, and its limited validity needs to be verified with more data and multiple constructs. An important research direction may be to use neuropsychological and behavioral instruments and psycholinguistic models to improve the measure’s validity. In addition, we would like to work on the long-term predictive power of the NVCQ for different situations with extended communication potential. Due to the general nature of the measure, we hypothesize that the NVCQ can be used in all communication domains, such as communication between clients and therapists, between bosses and employees, between teachers and students, and between family members and colleagues, to understand the role and influence of beliefs regarding non-verbal cues in effective communication between individuals.

### Practical implications

Finally, we suggest that the NVCQ can also be used in the real world to assess the role of non-verbal cues in communication. In academic settings, it can be used as a screening tool to identify students’ communication difficulties, such as difficulty in understanding the ole of maintaining eye contact or using appropriate gestures during presentations. Educators can also use it to assess the effectiveness of their non-verbal communication, for example, by evaluating how their understanding of facial expressions and body language impact on student engagement. In a professional context, the NVCQ could be a valuable resource for interpersonal communication training to identify strengths and areas for improvement, e.g., tone of voice in customer conversations or posture in meetings. HR departments could use the NVCQ in training workshops to improve teamwork and customer relations by focusing on the subtleties of non-verbal communication. In addition, the NVCQ can be used in research on non-verbal communication, for example, in examining its interaction with emotional intelligence or its role in therapeutic processes, thereby expanding theoretical and evidence-based understanding, especially with regard to cultural differences. Researchers could use the NVCQ to examine how non-verbal cues differ across cultures and the impact they have on cross-cultural communication. Healthcare professionals can also benefit from using the NVCQ to assess their communication skills or those of their clients. For example, it can help physicians assess their ability to convey empathy through facial expressions and gestures, thus promoting supportive interactions with patients. Therapists could use it to better understand their clients’ non-verbal cues, such as body language that indicates discomfort, creating a more empathetic and effective therapeutic environment.

## Conclusion

NVC (non-verbal communication) is an influential component of effective communication that provides skills important in all areas of life. In this paper, we documented the details of the NVCQ as part of a scientific investigation in psychology, and we developed the eight-item NVCQ self-report measure to assess individual differences in beliefs about or perceptions of NVC. Initial evidence for the psychometric potential of the NVCQ is encouraging and demonstrates the successful utility of the measure for all types of communication. We hope that the NVCQ will contribute to both future research in aforementioned directions and the understanding and demonstration of effective communication skills or skills training, as this is a central facet of successful human functioning.

## Data Availability

The datasets presented in this study can be found in online repositories. The names of the repository/repositories and accession number(s) can be found below: the data underlying this article are openly available in research box: https://researchbox.org/2479&PEER_REVIEW_passcode=YBRBCZ.

## Footnotes

^1^We assumed a unidimensional factor structure of the measure, but contrary to our preregistration, the two-factor structure emerged as the better one out of the three models we tested and reported in the earlier studies. Therefore, in this research, we proceeded with a two-dimensional structure of the scale.

^2^ https://aspredicted.org/blind.php?x=X8V_DR8
