# Supplementary material for: Non-verbal communication questionnaire: a measure to assess effective interaction
Source: Front Psychol. 2025 Jan 8;15:1409675. doi: 10.3389/fpsyg.2024.1409675 (PMC11750825; doi:10.3389/fpsyg.2024.1409675)
Supplement: Supplementary file 1 [file Table_1.DOCX]

**Supplementary Materials**

**Table S1**

Corrected Item-Total Correlations (Within Dimension) in Study 1 (*N* = 187), Study 2 (*N* = 202), Study 3 (*N* = 378) and Study 4 (*N* = 334)

| Items | Study 1 | | Study 2 | | Study 3 | | Study 4 | |
| --- | --- | --- | --- | --- | --- | --- | --- | --- |
|  | Corrected Item-Total Correlations | ω if item Deleted | Corrected Item-Total Correlation | ω if item Deleted | Corrected Item-Total Correlation | ω if item Deleted | Corrected Item-Total Correlation | ω if item Deleted |
| NV1 | .508 | .865 | .727 | .871 | .488 | .870 | .490 | .703 |
| NV3 | .741 | .814 | .513 | .817 | .754 | .812 | .566 | .677 |
| NV4 | .712 | .818 | .554 | .825 | .710 | .820 | .447 | .719 |
| NV5 | .718 | .816 | .731 | .819 | .724 | .815 | .495 | .703 |
| NV7 | .668 | .833 | .677 | .836 | .673 | .834 | .515 | .694 |
| NV2 | .409 | .734 | .401 | .724 | .392 | .706 | .288 | .288 |
| NV6 | .592 | .515 | .579 | .508 | .562 | .496 | .206 | .440 |
| NV8 | .550 | .555 | .542 | .544 | .525 | .532 | .290 | .277 |

**Auxiliary Analysis**

***Measurement invariance.*** In auxiliary analysis, to assess the psychometric equivalence of SNVQ across Pakistani (*n* = 609, studies 1-3) and Polish (*n* = 378, study 4) samples, we conducted a measurement invariance analysis using multigroup CFA (Kline, 2023). At first, we evaluated the model with two latent variables separately for both groups. Then, we assessed the configural invariance, where we assumed the same factor structure for both groups. Next, we assumed the factor loading to be the same for items to latent variables, called metric invariance, and lastly, we checked for scalar invariance, where we assumed equal intercepts for the items. We used model fit and change in fit indices (i.e., ΔRMSEA, ΔCFI, and ΔTLI) as the indicators for testing invariance.

The initial test of the model, having two latent variables, across Pakistani and Polish samples, maximum likelihood CFA yielded an acceptable model fit for all indices, RMSEA = .078, 90%CI [.066, .092], GFI = .989, CFI = .957, TLI = .937, SRMR = .034, which provides an initial support for configural invariance. We, therefore, conducted a formal test of measurement invariance between participant groups (Table S2 in supplementary materials). The values for ΔRMSEA, ΔCFI, and ΔTLI were below .01 as a result of configural, metric, and scalar invariance, which indicates the same structure of the scale, similar factor loadings of latent variables to items, and similar item intercepts across two country groups. Overall, we found that the measurement model was invariant across country groups for encouraging nonverbal cues (ENVC) and discouraging nonverbal cues (DNVC) while processing non-verbal communication. Thus, it seems valid to make cross-country comparisons regarding our scale scores.

We further found that Polish participants scored significantly higher (*M =* 5.15, *SD =* 0.81) than Pakistani sample (*M =* 3.47, *SD =* 1.48; Cohens’ *d* = -1.32) on NVCQ on the whole as well as on the ENVC (Polish: *M =* 5.35, *SD =* .89; Pakistani: *M =* 3.30, *SD =* 1.62; Cohens’ *d* = -1.47) and DNVC (Polish: *M =* 4.81, *SD =* 1.00; Pakistani: *M =* 3.76, *SD =* 1.59; Cohens’ *d* = -0.74) subscales.

**Table S2.** Measurement invariance across country groups in Study 4

| Type of invariance | RMSEA | GFI | CFI | TLI | ΔRMSEA | ΔCFI | ΔTLI |
| --- | --- | --- | --- | --- | --- | --- | --- |
| Configural | .078 | .989 | .957 | .937 |  |  |  |
| Metric | .081 | .987 | .948 | .933 | .003 | .009 | .004 |
| Scalar | .077 | .988 | .954 | .940 | -.004 | .006 | .007 |
